# Electrochemical determination of aripiprazole based on aluminium oxide nanoparticles modified carbon paste electrode

**DOI:** 10.55730/1300-0527.3523

**Published:** 2022-10-08

**Authors:** Funda ÖZTÜRK, Elif YÜKSEL, Pınar Esra ERDEN, Esma KILIÇ

**Affiliations:** 1 Department of Chemistry, Faculty of Science and Arts, Tekirdağ Namık Kemal University, Tekirdağ, Turkey; 2 Department of Chemistry, Polatlı Faculty of Science and Letters, Ankara Hacı Bayram Veli University, Ankara, Turkey; 3 Department of Chemistry, Faculty of Science, Ankara University, Ankara, Turkey

**Keywords:** Aripiprazole, square wave voltammetry, aluminium oxide nanoparticles, carbon paste electrode, pharmaceutical formulation, biological fluid

## Abstract

The electrochemical oxidation of aripiprazole was explored at a carbon paste electrode modified with aluminium oxide nanoparticles by cyclic voltammetry and square-wave anodic adsorptive stripping voltammetry. Experimental parameters such as carbon paste composition, scan rate, buffer pH, accumulation time, and accumulation potential were optimized in order to obtain high analytical performance. The incorporation of aluminium oxide nanoparticles into the carbon paste matrix enhanced the effective surface area of the carbon paste electrode and improved the sensitivity. On the aluminium oxide nanoparticles modified carbon paste electrode, aripiprazole exhibited an irreversible anodic peak at +1.17 V in pH 1.8 BR buffer solution. Under optimum conditions, the peak current exhibited a linear dependence with aripiprazole concentration between 0.03 and 8.0 μM with a detection limit of 0.006 μM. The analytical applicability of the voltammetric method was evaluated by quantification of ARP in human serum samples and pharmaceutical formulations.

## 1. Introduction

**Figure 1:**
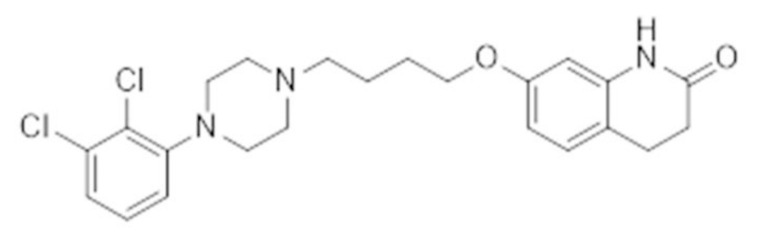
Chemical structure of ARP.

Aripiprazole (ARP): 7-{4-[4-(2,3-Dichlorophenyl)-1-piperazinyl]butoxy}-3,4-dihydro-2(1*H*)-quinolinone (Figure 1), is an atypical antipsychotic agent used to treat schizophrenia and other psychotic diseases [1,2]. Aripiprazole is considered a partial dopamine D2 and D3 receptor agonist, partial 5-HT1A receptor agonist, and 5-HT2A receptor antagonist [1,3]. Given the antidepressant properties of ARP which are helpful for the treatment of neurodegenerative diseases, the monitoring of this molecule in physiological fluids can be relevant for the effectiveness and the safety of a given treatment. Hence there is a need to develop an analytical method for the clinical monitoring and industrial quality control of ARP. Various techniques including capillary electrophoresis [3,4], high-performance liquid chromatography (HPLC) [5], HPLC–mass spectrometry (MS) [6] liquid chromatography–MS/MS [7,8], and spectrophotometry [9] were reported for the analysis of ARP. These techniques are commonly accurate and sensitive and show low detection limits [4,8]. However, most of these techniques have many disadvantages including high cost, long sample preparation time, unsuitability for on-site testing, and the need for expensive equipment and well-trained analyst. On the other hand, electroanalytical techniques offer practical, relatively less time-consuming, cost-effective, sensitive, and selective drug analyses when compared with other techniques [10]. The voltammetric determination of ARP from its dosage forms was investigated on bare glassy carbon electrode (GCE) [10,11] and graphene/TiO_2_/polyaniline modified GCE [12].

The interest in electrode modification for use in electrochemical sensing is growing rapidly. In this regard, working electrodes modified with nanomaterials offer many advantages over unmodified conventional solid electrodes including higher surface area, enhanced surface kinetics, better selectivity, higher sensitivity, lower detection limit, and better signal-to-noise ratio [13–15]. These electrodes find numerous applications in electrochemical sensing due to their aforementioned advantages. Metal oxide nanoparticles have been extensively used in electrochemistry applications such as electrochemical sensor and biosensors [16], lithium-ion batteries [17], and electrochemical capacitors [18] due to their chemical stability, low toxicity, good electrical conductivity, catalytic activity, high surface area and good biocompatibility [16, 19]. Aluminium oxide nanoparticles (Al_2_O_3_NP) have been used for electrode modification in the fabrication of electrochemical sensors [20–22].

Carbon paste electrodes (CPEs) have long been used in electrochemical sensing due to their wide anodic potential range, robustness, low residual current, surface renewability, chemical inertness, ease of fabrication, and low cost [23,24]. However, these electrodes also suffer from various shortcomings such as low sensitivity, slow electron transfer kinetics, and the need for higher over the potential for electrocatalytic process. The incorporation of metal oxide nanoparticles into the carbon paste matrix is a promising approach to overcome these limitations [24–27]. As far as we know, no voltammetric technique was reported using an Al_2_O_3_NP modified CPE for the quantification of ARP in biological fluids and formulations. In this study, electrochemical behavior of ARP was explored using an Al_2_O_3_NP modified CPE by cyclic voltammetry (CV) and square-wave anodic adsorptive stripping voltammetry (SWAAdSV). The effects of experimental conditions on the electrooxidation of ARP were also investigated. The development of a novel stripping voltammetric technique for the analysis of ARP in human serum and pharmaceutical formulations was another objective of the present study.

## 2. Experimental

### 2.1. Chemicals and samples

ARP, graphite powder (powder > 20μm), Al_2_O_3_NP (<50 nm particle size), glucose, paraffin oil, uric acid, lithium carbonate, human serum, potassium hexacyanoferrate(III), potassium hexacyanoferrate(II) and ascorbic acid were obtained from Sigma-Aldrich. Acetic acid, ethanol, phosphoric acid, potassium chloride, and boric acid were supplied from Merck. 0.04 M of each phosphoric acid, acetic acid, and boric acid were mixed for the preparation of Britton-Robinson (BR) buffer solution [28]. The stock solution of ARP (1.0 × 10^−3^ M) was dissolved in ethanol and diluted to the required concentration with pH 1.8, 0.04 M BR buffer. Pharmaceutical formulations (Abilify tablets) were obtained from a local drug store. Ten tablets containing 10 mg of ARP each were weighed and crushed to fine powder. A weight equivalent to one tablet was transferred into a flask and 50 mL ethanol was added. This mixture was sonicated for 1 h and then was centrifuged at 2000 rpm for 5 min and filtered through an ordinary filter paper. Clear supernatant liquor was transferred into a 100 mL volumetric flask and the volume was completed with ethanol to prepare the stock tablet solution. The required volumes from the stock solution were added to the electrochemical cell containing BR buffer.

Serum sample was stored in a freezer until the analysis. Methanol (1.0 mL) was added to a 1.0 mL serum sample to remove the proteins. The methanol–serum mixture was centrifuged at 2000 rpm for 5 min. The recovery of ARP was investigated using the supernatant. For this purpose, 10 μL of the supernatant was added to the electrochemical cell and the volume was completed to 10 mL with the supporting electrolyte (BR buffer, pH 1.8) and then the required volumes from ARP stock solution were transferred to the cell. After 5 min deaeration with nitrogen (99.99% purity), voltammetric measurements were carried out to determine the ARP content of the spiked serum sample.

### 2.2. Apparatus and measurements

Voltammetry studies were performed using a Bipotentiostat/Galvanostat Microstat 400 (Dropsens, Spain). A Gamry Instruments (Reference 3000) Potentiostat/Galvanostat was employed for the electrochemical impedance spectroscopy (EIS) studies. The working electrode was an Al_2_O_3_NP modified CPE (2.87 mm ID, BASi MF 2010). A platinum wire (0.5 mm diameter, BASi MW 1032) and an Ag/AgCl electrode (BASi MF 2052) were used as the auxiliary and reference electrode, respectively. pH values were measured with a HANNA Instruments HI 2211 pH meter using a combined glass electrode (Hanna Instruments, USA). Double distilled water from a PURELAB Option QDV 25 system was used throughout the experiments. All experiments were performed at room temperature.

EIS and CV experiments were conducted in a solution containing 0.1 M KCl and 5.0 mM K_3_Fe(CN)_6_/K_4_Fe(CN)_6_ (1:1) as the redox couple. The EIS spectra were obtained in the frequency range of 0.1–10000 Hz with an amplitude of 5 mV. CV studies were also carried out in BR buffer (pH 1.8) in the range of −0.50 to +0.80 V at 100 mV s^−1^ scan rate. SWAAdSV measurements were recorded in BR buffer at pH 1.8 on Al_2_O_3_NP–CPE. In SWAAdSV experiments, aliquots of ARP stock solution were added to the cell containing 10.0 mL of BR buffer. The cell content was deaerated with nitrogen for 5 min before the first measurement and 25 s between all individual measurements. The accumulation step was performed at 0.00 V for 300 s under stirring (300 rpm). Then the stirring was stopped and after 3 s rest period ARP was removed by stripping anodically using the SWV method. In SWV measurements a pulse-amplitude (Δ*E*_a_) of 0.01 V, frequency (*f*) of 10 Hz, and scan increment (Δ*E*_i_) of 0.01 V were used. The standard deviation of three measurements was used to draw the error bars in the figures.

### 2.3. Fabrication of the carbon paste electrodes

In order to prepare the Al_2_O_3_NP modified CPE 11.5 mg graphite powder, 3.5 mg Al_2_O_3_NP, and 10 μL paraffin oil were thoroughly hand mixed to obtain a homogeneous paste. After homogenization for 30 min, the carbon paste was filled into the hole of the electrode and smoothed on a weighing paper (Scheme). A similar procedure was used for the fabrication of the unmodified CPE (15 mg graphite powder and 10 μL paraffin oil).

## 3. Results and discussion

### 3.1. Optimization of modified carbon paste composition

**Figure 2:**
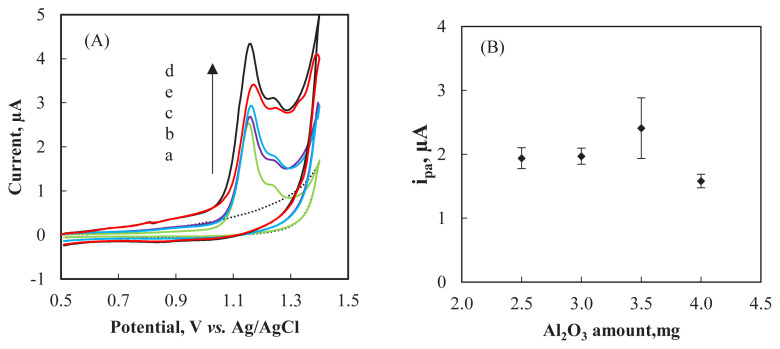
**(A)** Cyclic voltammograms of 2.0 ×10^−4^ M ARP solution in pH 1.8 BR buffer on bare CPE (a) and modified carbon paste electrodes containing different amounts of Al_2_O_3_NP (b: 2.5 mg; c: 3.0 mg; d: 3.5 mg; e: 4.0 mg Al_2_O_3_NP) scan rate 100 mVs^−1^, The dotted lines represent blank solution). **(B)** Effect of Al_2_O_3_NP amount on the anodic peak currents of ARP.

The influence of Al_2_O_3_NP amount on the ARP response of the CPE was investigated by CV in BR buffer containing 2.0 ×10^−4^ M ARP. The nanoparticle amount was varied between 2.5 and 4.0 mg while the total amount of graphite and nanoparticle kept constant at 15 mg and paraffin oil at 10 μL. Figure 2A shows the cyclic voltammograms for 2.0 × 10^−4^ M ARP solution in pH 1.8 BR buffer on bare CPE and modified carbon paste electrodes containing different amounts of Al_2_O_3_NP. The peak currents obtained at different Al_2_O_3_NP amounts are presented in Figure 2B. Peak current of ARP increased as the nanoparticle amount increased from 2.5 mg to 3.5 mg and decreased afterwards. The highest peak current was recorded at the Al_2_O_3_NP–CPE fabricated with 3.5 mg Al_2_O_3_NP. Therefore 3.5 mg was selected as the optimum nanoparticle amount for the modified carbon paste. The decrease observed after 3.5 mg Al_2_O_3_NP may be attributed to the limited diffusion rate of the substrate in the thicker films [29].

### 3.2. Electrochemical characterization

**Figure 3:**
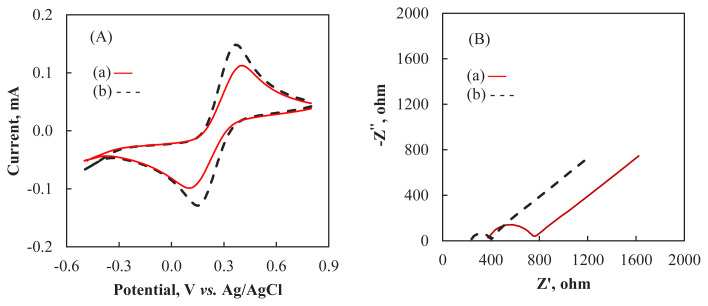
**(A)** Cyclic voltammograms (scan rate 100 mVs^−1^) and **(B)** Nyquist curves of 5.0 mM K_3_Fe(CN)_6_/K_4_Fe(CN)_6_ in 0.1 M KCl solution recorded at (a) CPE (solid line) and (b) Al_2_O_3_NP–CPE (dashed line).

Cyclic voltammograms of (a) bare CPE and (b) Al_2_O_3_NP–CPE recorded in a solution containing 0.1 M KCl and 5.0 mM K_3_Fe(CN)_6_/K_4_Fe(CN)_6_ are presented in Figure 3A. Well defined oxidation and reduction peaks corresponding to the redox couple are observed in both voltammograms. After the bare CPE (curve a) was modified with Al_2_O_3_NP (curve b), an increase of redox peak currents and a decrease of peak-to-peak separation (ΔEp = 190 mV) in comparison to the bare CPE (ΔEp = 262 mV) was observed. The higher peak currents and lower **ΔEp** value can be attributed to the presence of Al_2_O_3_NP in the carbon paste which can promote the transfer of electrons between the redox probe and the modified CPE [20]. Similar results were reported for Al_2_O_3_NP modified electrodes in the literature [30].

EIS is a valuable method to investigate the interfacial characteristics of modified electrodes. Impedance spectra can be presented in the form of Nyquist plots which reveal electron transfer kinetics and diffusion characteristics. In a typical Nyquist plot, the diameter of the semicircle is equivalent to the charge transfer resistance (*R*_ct_) [31]. Figure 3B depicts the Nyquist plots of Al_2_O_3_NP–CPE and bare CPE recorded in a solution containing 0.1 M KCl and 5.0 mM K_3_Fe(CN)_6_/K_4_Fe(CN)_6_. The Al_2_O_3_NP–CPE (curve b) revealed a smaller semicircle than bare CPE (curve a), indicating a lower electron transfer resistance due to the incorporation of Al_2_O_3_NP into the carbon paste matrix.

### 3.3. Electrochemical behavior of aripiprazole

The electrochemical behavior of ARP at bare CPE and Al_2_O_3_NP–CPE was explored in BR buffer (pH 1.8) in the presence of 2.0 ×10^−4^ M of ARP (Figure 2A). A well defined oxidation peak was obtained at bare CPE and Al_2_O_3_NP–CPE at a potential of +1.15 V and +1.17 V, respectively. On the other hand, no cathodic peak was obtained at the reverse scan suggesting a totally irreversible electrode reaction. The oxidation peak current of ARP was higher at Al_2_O_3_NP–CPE compared with that of the peak at the bare CPE. The significant increase in peak current indicates a higher electron transfer rate for ARP at Al_2_O_3_NP–CPE which can be ascribed to the larger effective surface area of the modified electrode.

The effective surface area of electrodes was investigated using Randles–Sevcik equation:


(1) ip=(2.69×105)n3/2 D1/2ν12AC

where, *i*_p_ is the peak current (A), *n* is the number of electrons transferred, *A* is the electroactive surface area (cm^2^), *D* is the diffusion coefficient of K_3_[Fe(CN)_6_] in the solution (7.6 × 10^−6^ cm^2^ s^−1^), *C* is the concentration of K_3_[Fe(CN)_6_] (mol cm^−3^) and *v* is the scan rate (V s^−1^) [32]. The effective surface areas were calculated to be 0.1 cm^2^ and 0.126 cm^2^ for bare CPE and Al_2_O_3_NP–CPE, respectively.

#### Influence of scan rate

**Figure 4:**
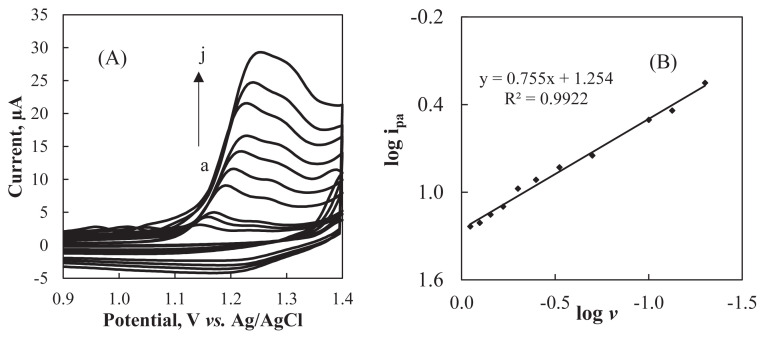
**(A)** Cyclic voltammograms of 2.0 × 10^−4^ M ARP at different scan rates in pH 1.8 BR buffer (scan rates: a: 0.05, b: 0.075, c: 0.1, d: 0.2, e: 0.3, f: 0.4, g: 0.5, h: 0.6, i: 0.7 and j: 0.8 Vs^−1^). **(B)** Plot of log i_pa_ vs.log *v.*

In order to enlighten the nature of the electrochemical reaction that occurs at the surface of Al_2_O_3_NP–CPE, the influence of scan rate (*v*) on peak potential (*E*_pa_) and anodic peak current (*i_pa_*) was tested using CV. Figure 4A depicts that the peak potentials shifted to more positive potential values as the scan rate increased from 0.05 to 0.80 Vs^−1^. This result shows that the electrooxidation step is not reversible [33].

The relationship between the logarithm of peak current and the logarithm of scan rate (Figure 4B) can be presented by the corresponding equation: log (*i_pa_*) = 0.755 log *v* + 1.254 with R^2^ = 0.9922 [34]. The obtained slope (0.755) is close to the theoretical value (1.0) for the adsorbed species. Therefore, the nature of the electrochemical reaction for the oxidation of ARP at Al_2_O_3_NP–CPE can be assigned to the adsorption controlled electrode process [35,36].

The diffusion coefficient of ARP was estimated from the following equation validated by Garrido for the adsorption phenomena [37].


(2) $$i_{p}=1.06\times 10^{6}n^{2}ACvD^{1/2}t_{p}^{1/2}$$

In this equation, *i_p_* is the oxidation peak current (A), *A* is the area of electrode surface (cm^2^), *D* is the diffusion coefficient (cm^2^s^−1^), *C* is the analytical concentration of diffused species (molcm^−3^), *t_p_* is the time required to reach peak potential from the beginning of potential scan (s), *n* is the number of electrons transferred and *v* is the scan rate (Vs^−1^). The mean of the diffusion coefficient was calculated as (1.19 ± 0.67) × 10^−8^ cm^2^s^−1^.

#### Influence of buffer pH

**Figure 5:**
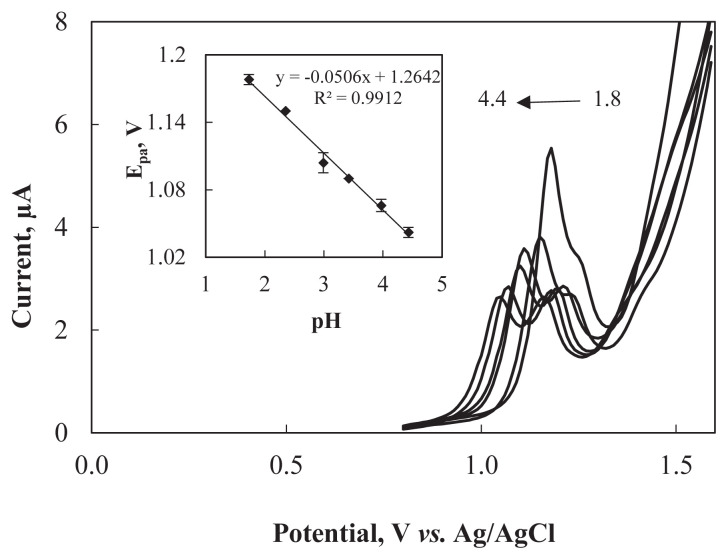
Square wave voltammograms of 2.0 × 10^−6^ M ARP at Al_2_O_3_NP–CPE in BR buffer with different pH values (1.8; 2.4; 3.0; 3.4; 4.0; 4.4). Inset: plot of *E*_pa_ vs. pH.

The pH of the working buffer might affect the electrode reaction. Thus, the influence of pH on the electrochemical behavior of ARP on Al_2_O_3_NP–CPE was explored by SWV in a pH range of 1.8–4.5. Figure 5 shows that the anodic peak potential shifted to more negative values and peak current decreased with the increase in pH of the buffer solution. In SWV studies, the maximum current response for oxidation of ARP was achieved at pH 1.8, and poor peak shapes were obtained after pH 4.5 (Figure 1). Therefore, pH 1.8 was used as the optimum pH value in the further studies.

The linear equation of *E*_pa_ *vs.* pH (Figure 5 inset) is expressed as follows: *E*_pa_ (V) = 0.0506 pH–1.2642 with R^2^ = 0.9912. The value of the slope (0.0506) is close to the theoretical value (0.0592 V) indicating that equal numbers of protons and electrons are released in the electrooxidation of ARP [38,39]. The slope of this relation was evaluated in the following equation [40]:


(3) $$E_{p}=E^{0}+\frac{RT}{nF}\;\text{ln }\frac{ox}{Red}-\frac{\partial RT}{nF}\;\text{ln }H^{+}$$

Where *E*^o^ is standard peak potential in V, ∂ is the number of protons involved in the reaction, *Ox* and *Red* are equilibrium concentrations of oxidized and reduced species in M and the *n, F, R* and *T* have their usual meanings [41]. The ratio of proton to electron involved in the mechanism was found to be 0.85. The peak potential shifts to less anodic values with an increase in pH which indicates that electron transfer is accompanied by proton transfer [42].

#### Proposed mechanism

The clarification of the mechanism of electrochemical oxidation of ARP on the Al_2_O_3_NP–CPE has been beyond the major goal of this study. However, the experimental results show that in BR buffer at pH 1.8, the oxidation of ARP is adsorption controlled and not reversible. The electrochemical process involves equal numbers of protons and electrons. Figure 2A shows that the relationship between *i_p_*/*v*^1/2^ *vs.* log *v* was not linear which also suggested that the process on the electrode surface was controlled by adsorption. The linear relation between peak potential and logarithm of scan rate can be presented by the following equation, *E*_pa_ (mV) = 87.095 log *v* (mVs^−1^) + 993.41 (R^2^ = 0.9754) (Figure 2B) which is in good agreement with the EC mechanism, where the electron transfer at the electrode is followed by an irreversible chemical reaction [10,43]. The proposed mechanism is consistent with the results reported on the oxidation process of ARP on GCE [10].

### 3.4. Analytical performance of the voltammetric method

In this study, the analytical determination of ARP was performed by SWAdASV using the presented Al_2_O_3_NP–CPE. For this purpose, the experimental parameters related to the ARP determination using the SWAdASV method were subjected to optimization. A constant concentration of ARP (2.0 μM) was used in the optimization studies.

**Figure 6:**
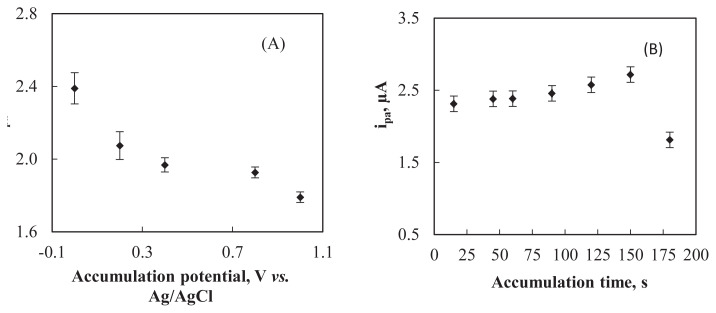
Effects of **(A)** accumulation potential and **(B)** accumulation time on the anodic peak current of 2.0 × 10^−6^ M ARP on Al_2_O_3_NP–CPE.

In the adsorptive stripping voltammetry, the effect of accumulation potential and accumulation time on the SWAAdSV signal were explored. The effect of accumulation potential on the oxidation current of ARP at Al_2_O_3_NP–CPE was studied in the potential range of 0.0 V–1.0 V (Figure 6A). The peak current decreases at more positive accumulation potentials after 0.0 V and the maximum peak current in the accumulation step was obtained at 0.0 V. Thus, an accumulation potential of 0.0 V was adopted for further studies. The effect of accumulation time on peak current was also investigated in the range of 15–180 s. The peak current increased as the accumulation time increased from 15 to 150 s and decreased afterwards (Figure 6B). The optimum accumulation time was selected as 150 s.

**Figure 7:**
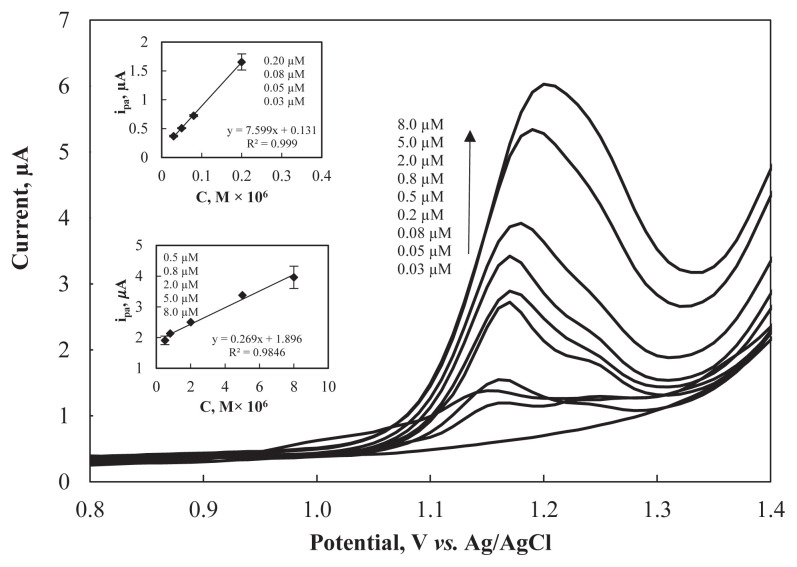
Square wave voltammograms of ARP at Al_2_O_3_NP–CPE containing different concentrations of ARP ranging from 0.03 μM to 8.0 μM (inset: the corresponding calibration curves).

Under the optimum working conditions, various concentrations of ARP (0.03 μM–8.0 μM) were used to plot the calibration curves by SWAAdSV measurements (Figure 7). As shown in Figure 7 inset, the presented method displayed two linear ranges for ARP determination at the Al_2_O_3_NP–CPE. For the first range (from 0.03 μM (0.013 mg L^−1^) to 0.2 μM (0.090 mg L^−1^) the regression equation was: ip(μA) = 7.599 × *C*_ARP_ (μM) + 0.131 (R^2^ = 0.999). For the second range (from 0.5 μM (0.224 mg L^−1^) to 8.0 μM (3.59 mg L^−1^)) the regression equation was: ip(μA) = 0.269 × *C*_ARP_ (μM) + 1.896 (R^2^ = 0.9846).

The detection limit (LOD) and quantification limit (LOQ) values were evaluated by the following formula: LOD = 3*s*/*m* and LOQ = 10*s*/*m* [44], using the standard deviation of the intercept (*s*) and the slope of the regression line (*m*). The LOD and LOQ values were calculated as 0.006 μM (0.0027 mg L^−1^) and 0.019 *μ*M (0.0085 mg L^−1^), respectively.

In order to validate the presented method, parameters such as accuracy, stability, precision, reproducibility, and selectivity were also determined. Five Al_2_O_3_NP–CPEs were prepared using the same procedure and the response of these modified electrodes to 2.0 ×10^−6^ M ARP was recorded to study the reproducibility of the presented method (Figure 3A). The relative standard deviation (RSD%) of the current response was found to be 2.9%. This result shows the high reproducibility of the presented method. The repeatability of ARP in a BR at pH 1.8 was studied under optimal experimental conditions by recording the changes in the anodic peak current of a 2.0 ×10^−6^ M standard ARP solution (Figure 3B). The RSD of peak currents for five replicates of measurements was found to be 1.8%. The result indicates good repeatability of the sensor. In order to explore the long-term stability of the Al_2_O_3_NP–CPE, the electrode was placed in pH 1.8 BR buffer containing 2.0 × 10^−6^ M ARP and voltammograms were recorded for a period of 10 days at intervals of 3 days. After 10 days the peak current decreased by 8%, which showed that the sensor had satisfactory stability.

The selectivity of the presented method was investigated in the presence of common interferences such as ascorbic acid, glucose, and uric acid. pH 1.8 BR buffer solutions containing 0.054 μM ARP and 50 μM of the potential interfering species were analyzed by means of the presented method (Table 1). From the selected potential interfering species, none were found to interfere seriously with the electrochemical sensing of ARP. The tolerance limit was less than ± 5.5% for each interference. Therefore, the proposed method can be considered to be specific.

The analytical performance characteristics of the presented method on the determination of ARP were compared with the previous studies. Table 2 shows that the presented method exhibits a lower detection limit and wider linear range than most of the previously reported papers.

### 3.5. Real sample analysis

The performance of Al_2_O_3_NP–CPE as a novel electrochemical sensor for the analysis of ARP was tested in serum samples and pharmaceutical formulations. Table 3 presents the results for the serum sample and pharmaceutical formulation. The accuracy of the Al_2_O_3_NP–CPE sensor was evaluated by its recovery values. The direct calibration method was used in real sample analysis. The data presented in the tables indicate that average recovery values are in good agreement and the RSD values are below 6%. The recovery data confirmed the ability of Al_2_O_3_NP–CPE as a sensitive sensor for the analysis of ARP in real samples.

In order to evaluate the accuracy of the described procedure, the recovery of a constant concentration of ARP was calculated at optimum experimental conditions. Recovery values range between 104.83%–106.30% for tablets and 102.88%–106.83% for serum analysis. These values indicate that the presented method is highly accurate and can be used in the determination of ARP in tablet samples and human serum. The performance of the presented method was also evaluated by the t test. The results given in Table 3 indicate that the *t*_exp._ values do not exceed the *t*_crit._ (4.30 for two degrees of freedom at a 95% confidence level) value confirming that the results of the voltammetric method and the spiked amount show no difference at a confidence level of 95%. It can be concluded that the presented method could be an alternative to the methods reported in the literature for ARP analysis. Three replicate measurements of different ARP solutions were evaluated to calculate the precision of the presented method. The precision of the presented method is excellent with an RSD of the recovery values ranging from 2.47% to 5.24% for all experiments (Table 3).

## 4. Conclusion

A novel simple and sensitive voltammetric method, based on Al_2_O_3_NP modified CPE was described for the determination of ARP and the electrochemical oxidation of ARP was studied on this electrode for the first time. The electrochemical reaction for the oxidation of ARP at Al_2_O_3_NP–CPE is an irreversible and adsorption controlled electrode process. The presented method provides a rapid, highly sensitive, selective, low cost and simple approach for the analysis of ARP in tablets and spiked human serum sample. Wide linear range (0.03–8.0 μM), low detection limit (0.006 μM), excellent repeatability (RSD = 1.8%), good reproducibility (RSD = 2.9%) and satisfactory stability are the other advantages of this sensor. The presented method also offers the advantage that no complex sample preparation steps are required in a serum sample. It can be concluded that the presented method can be an applicable method for analytical purposes.

## Figures and Tables

**Scheme:**
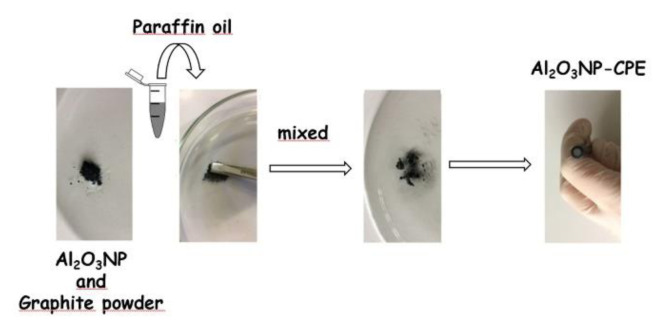
Fabrication procedure of Al_2_O_3_NP-CPE.

**Table 1:** Effect of interferences on the voltammetric determination of ARP.

Potential interfering species	Concentration of Interference, μM	Concentration of ARP, μM	% Interference
Uric acid	50	0.054	−4.40%
Ascorbic acid	50	0.054	−5.43%
Glucose	50	0.054	−4.72%

**Table 2:** Comparison of the presented method with the previous voltammetric studies.

Method	Working electrode	Linear range, M	LOD, M	Recovery tablet %	Recovery serum %	Recovery urine %	Ref.
LSV AdSV	GCE	2.2 × 10^−7^–1.1 × 10^−5^ 8.9 × 10^−9^–8.9 × 10^−8^	1.1 × 10^−7^–2.2 × 10^−9^	-	–	95–103 94–106	[10]
DPV SWV DPAAdSV SWAAdSV	GCE	1.14 × 10^−5^–1.57 × 10^−4^ 2.2 × 10^−7^–1.36 × 10^−5^	6.2 × 10^−6^ 5.5 × 10^−6^ 1.4 × 10^−7^ 1.1 × 10^−7^	98.8 97.4 - -	97.2 98	97 101.4	[11]
SWV	GRP/TiO_2_/PANI/GCE	1.1 × 10^−8^–8.9 × 10^−8^	5.2 × 10^−9^	99–101.6	–	–	[12]
SWAAdSV	Al_2_O_3_NP–CPE	3.0 × 10^−8^–2.0 × 10^−7^ 5.0 × 10^−7^–8.0 × 10^−6^	6.0 × 10^−9^	104.8–106.3	102.8–106.8	– –	present work

GRP: graphene, PANI: polyaniline, LSV: linear sweep voltammetry, AdSV: adsorptive stripping voltammetry, DPV: differential pulse voltammetry, DPAAdSV: differential pulse anodic adsorptive stripping voltammetry

**Table 3:** Results of Al_2_O_3_NP–CPE sensor performance in spiked human serum and tablets.

Sample	Spiked, μg	Found, μg	Recovery*, %	RSD, %	*t*_exp._
Serum	0.15	0.155; 0.160; 0.148	102.88 ± 4.01	3.90	1.24
	5.52	6.241; 5.808; 5.642	106.83 ± 5.60	5.24	2.11
	Nominal value, mg	Found, mg	Recovery*, %	RSD, %	*t*_exp._
Tablet	10	10.10; 10.41; 10.94	104.83 ± 4.25	4.05	1.97
	10	10.52; 10.93; 10.44	106.30 ± 2.63	2.47	4.15

* Results of recovery values are given as mean ± *ts*/√N (at 95% confidence level).
